# High Occurrence Among Calves and Close Phylogenetic Relationships With Human Viruses Warrants Close Surveillance of Rotaviruses in Kuwaiti Dairy Farms

**DOI:** 10.3389/fvets.2022.745934

**Published:** 2022-03-08

**Authors:** Mohammad A. Alotaibi, S. Al-Amad, Ali Chenari Bouket, H. Al-Aqeel, E. Haider, A. Bin Hijji, Lassaad Belbahri, Faizah N. Alenezi

**Affiliations:** ^1^Biotechnology Program, Environmental and Life Sciences and Research Center, Kuwait Institute for Scientific Research, Kuwait City, Kuwait; ^2^East Azarbaijan Agricultural and Natural Resources Research and Education Center, Plant Protection Research Department, Agricultural Research, Education and Extension Organization (AREEO), Tabriz, Iran; ^3^Laboratory of Soil Biology, University of Neuchatel, Neuchatel, Switzerland; ^4^Jaber Al-Ahmad Armed Forces Hospital, Kuwait City, Kuwait

**Keywords:** rotavirus, Kuwait, calves, RNA, phylogenetic RVA

## Abstract

Rotavirus, one of the main pathogens causing morbidity and mortality in neonatal dairy calves worldwide, is responsible for 30–44% of cattle deaths. It is considered to be the most common etiologic agent of diarrhea in neonatal dairy calves and children, the dominant type being group A. Two hundred seventy animals from 27 farms from 2 regions of Kuwait were tested for the presence of Rotavirus serogroup A (RVA) using latex agglutination test (LAT) and reverse transcription–polymerase chain (RT-PCR) testing. RVA non-structural proteins NSP1-2, NSP4-5 and capsid protein genes VP1-7 were characterized by next generation sequencing. LAT was positive in 15.56% of the animals, and RT-PCR in 28.89%. Using RT-PCR as a reference method, LAT was 100% specific but only 83.33% sensitive. ANOVA analysis showed correlation only with the location of the farms but no significant correlation with the age and sex of the animals. Although there was a tendency of clustering of RVA positive animals, it did not reach statistical significance (*p* = 0.035 for LAT). The phylogenetic analysis showed that Kuwaiti isolates of group A rotavirus clustered with human rotaviruses. Taken together, it seems that rotavirus was present in most of the dairy farms in Kuwait. The high occurrence of the virus in calves in Kuwaiti dairy farms and the close phylogenetic affinity with human isolates warrants urgent action to minimize and control its spread between calves in farms.

## Introduction

Neonatal and young calve's diarrhea (NCD) is a common syndrome and major health issue in the livestock industry, worldwide, resulting in both short- and long-term economic consequences that stem from increased morbidity and mortality. Decreased growth rate, increased caring needs, prolonged calving time, and incidental death are the main causes of the substantial increase in the overall costs of treatment and present a serious obstacle for a sustainable livestock industry. Given that in many countries this industry is a major economic resource, the constant monitoring of livestock health is of paramount importance. Moreover, as the industry is constantly growing due to the high demand for cattle meat, milk, and milk products, the requirement for verified, consistently healthy animals is increasing.

The main etiological agent for bovine diarrhea is rotavirus A (RVA), a species of a double stranded RNA virus family, the *Reoviridae*, that acts and transmits through the gastrointestinal route. Full genomic classification has revealed a common origin for porcine, bovine and human rotavirus strains (1, 2). As with all zoonoses, the virus moves back and forth from the human caregivers to the animals and vice versa, creating a constant open pool of new infections (3). Furthermore, the transmission within the same farm imposes the immediate and timely sequestration of the infected animals, before creating viral outbreaks that affect the whole herd. Moreover, there is evidence of interspecies transmission, such as from goat to cattle (4).

RVA infections are a constant health issue across the cattle farming industry, worldwide. A decade-long study, in Brazil, revealed that the RVA cattle infection rate remains constant through the years, despite the progress in our understanding of health epidemiology, the worldwide compliance clauses and the rigorous animal testing (5). In Uruguay, a large dairy and beef producer, RVA infection occurrence in calves reaches 57%. Additionally, there was a statistically significant difference between dairy (59.5%) and beef calves (28.4%), while diarrhea was associated with higher viral load (6). In Northwest Argentina the reported occurrence was 63% in dairy farms, while in beef herds the occurrence was 40% (7). In Bangladesh, RVA RNA was detected in only 6.2% of all cattle tested (8), while in India the reported RT-PCR positivity was 10.19% (9).

In Kuwait, the Public Authority of Agriculture and Fisheries (PAAF), reports approximately 9,004 dairy cattle, with a production rate of 19.78 liters/cow (10). RVA infection has been constantly impacting severely the Kuwaiti livestock, with a mean calf mortality of almost 44% (11). Indeed, in previous RVA outbreaks, the mortality rate reached almost 90% in some farms (12, 13), with a cost of $252 per year per dead calf (14).

The present study aims to assess the current occurrence of rotavirus A infection in Kuwaiti cattle livestock, using the routine diagnostic tools that are used in real life situations (namely agglutination and RT-PCR), as well as novel next generation sequencing (NSG). We believe that this study will update the current situation of livestock in the country, help promote RVA surveillance and awareness campaigns and assist animal health authorities and policy makers draw up relevant guidelines and recommendations.

## Materials and Methods

### Sampling

Fecal samples (triplicates) from 10 random calves, each <1-year old, were collected from 27 randomly selected farms from two regions, namely 25 from Sulaibiya (north) and 2 from Kabed, during a period of 12 months (January-December 2016), resulting in a total of 810 samples. Sulaibiya is a major livestock region, where 47 dairy farms and the Kuwait Cattle Research Centre of the Public Authority for Agricultural Affairs, and Fish Resources are located.

Samples of calve's feces were collected, randomly, regardless of the calf health condition or the presence/absence of any symptoms. The total number of calves in dairy farms was 852. Upon which the samples size was calculated to be around 265 and was rounded to 270 to test 10 calves from each farm so that we could achieve a statistical accuracy of 95% and a confidence interval of +/−3.45%, was calculated (15, 16).

### Latex Agglutination

Approximately 10 g of calf feces was suspended in 5 ml of phosphate buffered saline solution, mixed by vortexing 30 sec, then 2 drops of fecal suspension were placed into a specific well, in order to perform latex agglutination (LAT) RVA detection, using the LTA kit Virotect-Rota, (Omega Diagnostics Ltd, UK), according to supplier instructions.

### RNA Extraction

RNA was extracted using QIAamp Viral RNA Mini Kit (QIAamp Viral RNA Mini Kit, 52906, Qiagen, UK). Viral RNA extraction was performed according to the manufacturer and has been previously reported (17). The total extracted viral RNA was quantified spectrophotometrically by Nano-drop (18) to determine purity and concentration, then aliquoted and stored at−80°C, until further use.

### RT-PCR

RT-PCR amplification was performed to detect RVA capsid VP7 RNA sequences, using the primers previously described. RT-PCR was first optimized to ensure the highest sensitivity (17). Briefly, 2 μl of RNA extract were reverse transcribed and amplified in a 25 μl one-step reaction for 35 cycles. The resulting amplicon was expected to be 257 bp, and it was visualized by gel electrophoresis, using 1.2% gel agarose with 0.05% ethidium bromide.

### NGS of the RVA

For NGS, the non-structural proteins NSP1-2, NSP4-5 and capsid protein genes VP1-7 were selected. RNA sample concentration was adjusted to 100 ng. An RNA library was then generated using NEBNext® Ultra RNA Library Prep Kit for Illumina (New England Biolabs, UK) following manufacturer recommendations. After library quality assessment using a Bioanalyser (Agilent Technologies, UK), sequencing was carried out on a MiSeq sequencer (Illumina, UK). MiSeq reporter software (Illumina, UK) was then used to mine the generated data. Reads were aligned with the sequence of targeted rotavirus non-structural proteins NSP1-2, NSP4-5 and capsid protein genes VP1-7 and consensus sequences of the protein genes used as reference sequences in CLC Genomics Workbench (CLC bio, UK). The assembled gene sequences were compared to reference rotavirus gene sequences using the ClustalW program within MEGA 5.05 package (19). Using PhyML version 3.0, phylogenetic trees were constructed (20) and p-distances were calculated in MEGA 5.05 to determine the similarities of the genes to reference strains in GenBank.

### Statistical Analysis

Sensitivity, specificity, positive (PPV) and negative (NPV) predictive values were calculated as described at https://www.medcalc.org/calc/diagnostic_test.php.

The data were analyzed using the free access statistical software Jamovi 1.8.2.

## Results

### Study Population

The characteristics of the participating animals are summarized in Table 1. The animal's ages ranged from 7 to 150 days with a mean of 45.03 days. Since these are dairy farms, most animals were female (*n* = 209) with only 61 males.

**Table 1:** RVA infection in male and female calves, by LTA and RT-PCR.

	**Age (days)**				
**Farm no**	**Range**	**Mean**	**F/M Ratio**	**LTA (+)**	**RT-PCR (+)**
1	25–90	42.0	8/2	0	3
2	30–90	51.0	8/2	0	3
3	20–120	51.0	8/2	0	0
4	20–90	54.5	8/2	1	5
5	10–150	57.0	10/0	2	4
6	20–150	72.0	10/0	1	3
7	20–25	22.0	10/0	7	10
8	8–60	25.5	5/5	10	10
9	20–60	32.0	9/1	0	1
10	20–60	34.5	9/1	0	2
11	20–75	37.0	7/3	0	0
12	7–75	36.7	7/3	0	1
13	20–75	35.5	6/4	0	0
14	15–69	44.6	6/4	1	1
15	15–75	55,0	8/2	0	0
16	7–150	38.8	9/1	0	0
17	30–120	68.0	6/4	0	0
18	20–50	37.0	10/0	0	0
19	30–90	68.0	10/0	0	0
20	30–75	54.5	10/0	0	0
21	15–30	23.7	3/7	0	0
22	15–45	25.5	9/1	0	0
23	15–90	53.5	7/3	0	0
24	25–45	36.0	6/4	0	6
25	10–30	21.5	8/2	4	8
26	20–150	61.5	8/2	9	10
27	30–120	77.5	4/6	7	10
Total			209/61	42	78

### Presence of RVA Viral Infection

**Figure 1:**
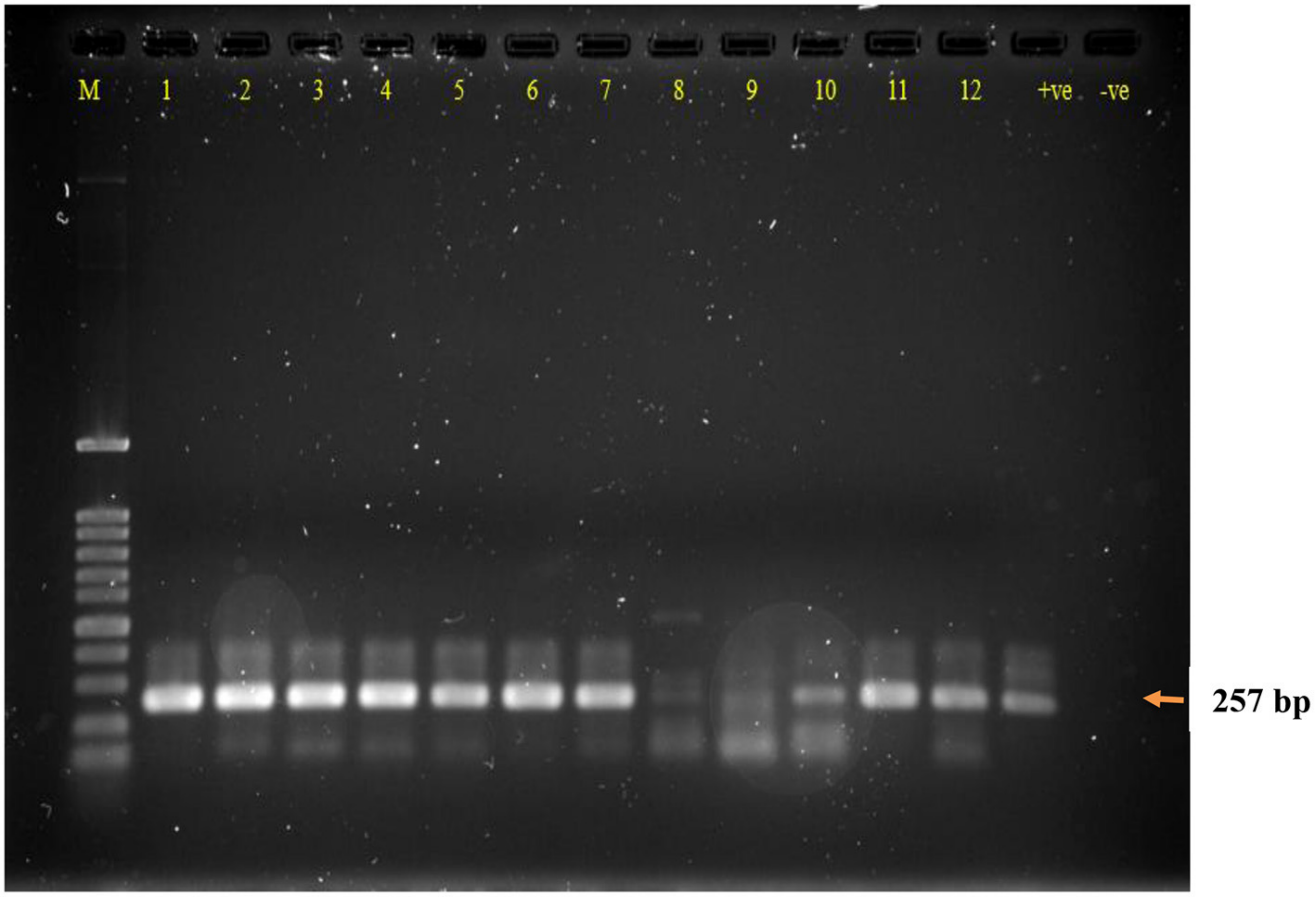
Agarose gel electrophoresis of RT-PCR DNA product of calve's fecal samples following RNA extraction and controls. *Lane M: DNA ladder 100 bp, Lanes 1–12: samples; (*+*ve) RT-PCR RVA positive control; (*−*ve) RT-PCR negative control. RT-PCR dsDNA product size 257 bp*.

Out of the 270 animals 42 were positive for LAT (15.56%), almost double were positive for RT-PCR (28.89%). All LAT positive samples were RT-PCR positive, accounting for a strong correlation (*p* < 0.001), but the reverse does not apply. All triplicates showed the same result; thus, reproducibility of both LAT and PCR is 100%. Figure 1 illustrates an example of RT-PCR for 12 samples.

Considering PCR as a reference method, the LAT sensitivity was 83.33% (73.62 to 90.58%) and specificity is 100.00% (97.97 to 100.00%). The PPV was 100.00%, while the NPV was 93.66%. Therefore, the accuracy of LAT was 95.19%.

Another observation from Table 1 is that RVA positive cattle are clustered in specific farms at least as far as the LAT test is concerned. However, there seems to be no correlation with individual farms (*p* = 0.035 for LAT; *p* = 0.264 for RT-PCR), although it seems that the farm location is significant (*p* < 0.001 for both LAT and RT-PCR), with Kabed being associated with higher viral presence (Table 2). The small number of Kabed farms mandates that this correlation should be further tested and not be generalized. Moreover, sex is not correlated with RVA infection (*p* = 0.027 for LAT; *p* = 0.446 for RT-PCR). Age also does not correlate with RVA infection (*p* = 0.129 for LAT; *p* = 0.133 for RT-PCR).

**Table 2:** Non-parametric ANOVA correlation statistics (Kruskal-Wallis).

	**LTA (+)**		**RT-PCR (+)**	
	***x^**2**^***	***p*-value**	***x^**2**^***	***p*-value**
Age	2.30	0.129	2.261	0.133
Sex	4.88	0.027	0.581	0.446
Farm Code	4.45	0.035	1.245	0.264
Farm Site	68.04	<0.001	52.972	<0.001

### Phylogenetic Analysis of Kuwaiti RVA Strains

All generated sequences have been submitted to GenBank (MH717372, MH717373, MH717374, MH717375, MH717366, MH717367, MH717368, MH717369, MH717370 and MH717371) (Table 3). Blast analysis of rotavirus non-structural proteins NSP1 clearly showed strong similarity with RVA NSP 1 sequences from Ireland (GQ428136), Turkey (KX212886), Thailand (LC367320) among numerous other matches.

**Table 3:** Rotavirus group A Kuwaiti isolate gene sequences and their accession numbers.

**GenBank accession number**	**Gene location**
MH717372	NSP1 gene, partial cds
MH717373	NSP2-like gene, partial sequence
MH717374	NSP4-like gene, partial sequence
MH717375	NSP5-like gene, complete sequence
MH717366	VP1 capsid protein gene, partial cds
MH717367	VP2 capsid protein gene, partial cds
MH717368	VP3 capsid protein gene, partial cds
MH717369	VP4 capsid protein gene, partial cds
MH717370	VP6 capsid protein gene, partial cds
MH717371	VP7 capsid protein gene, partial cds

**Figure 2:**
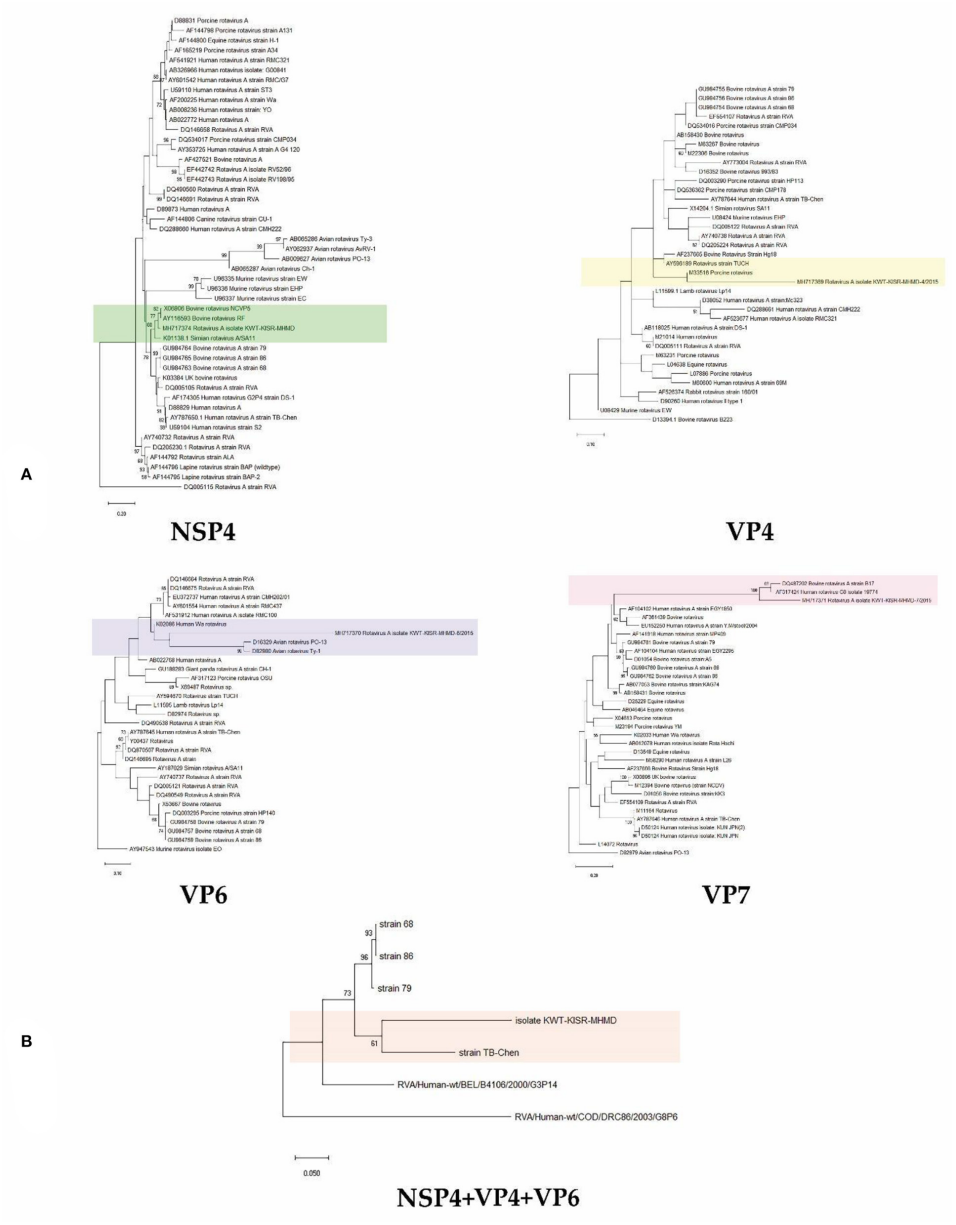
Neighbour-joining phylogenomic tree of veterinary important viruses including KWT-KISR-MHMD isolate **(A)** using NSP4, VP4, VP6 and VP7 genes separately and **(B)** concatenated (NSP4+VP4+VP6) genes. DQ005115, D13394.1, AY947543, D82979 and RVA/Human-wt/BEL/B4106/2000/G3P14 were used as outgroups, respectively. Supports for branches were assessed by bootstrap resampling of the data set with 1000 replications.

Phylogenetic analysis of Kuwaiti isolates of rotavirus group A gene sequences using either non-structural protein NSP4 or capsid protein genes VP4, 6 and 7 in single gene trees allowed confirmation that Kuwaiti isolates of rotavirus group A cluster with rotavirus group A isolates collected around the world. NSP4, VP4 and VP7 sequences showed close relationships with bovine isolates of the rotavirus, while NSP4, VP4, VP6 and VP7 additionally suggest close relationships with rotavirus isolates of simian, porcine, avian and human origins. Phylogenetic analysis using concatenated NSP4, VP4, VP6 and VP7 gene sequences unambiguously documented the close relationship of the Kuwaiti isolates with rotaviruses of human origin (Figure 2). The sequencing was performed for 10 samples from the 2 farms in Kabed and the 8 from the Saulibiya were sequenced, that covered all locations.

## Discussion

Rotavirus in calves in Kuwait represents a major challenge to dairy producers, since it is one of the contributing reasons that leads to a high rate of economic losses, e.g., in Kuwait $252 per year per dead calf (14). The livestock industry is growing in Kuwait due to high domestic demand for cattle meat, milk and milk products. The annual production of milk is 4,500 l/cow and the total number of cattle in Kuwait is 27,000 (12). Calf mortality is one of the major limiting factors for the sustainable growth of livestock industry, and sometimes the mortality rate could reach up to 90% in some farms in Kuwait (12). Infectious diseases are a major cause of mortality. Rotavirus is a high-risk pathogen if not treated well in advance, and calf mean mortality in Kuwait was found to be 44% (11, 14).

In Kuwait, a study of calf mortality was performed in 2001 by Razzaque et al. (14) who reported high mean calf mortality rates as follows: 44% in preweaned calves, while in some improperly managed farms, the mortality rate reached 90%. The high mortality rate of calves, which led to a livestock and financial loss for the farmers, was shown to be due to pathogens, detected using immunological methods, causing diarrhea, pneumonia, and dehydration in calves. The detected pathogens included rotavirus as well as bacteria (*Salmonella, E*. *coli*, and *Pasteurella*). The calculated financial loss in Kuwait per dead calf was $252 (14). The study also indicated that the farmers do not provide proper protection for their calves, such as vaccination, or use biosecurity measures, such as the isolation of infected calves. The lack of these measures has led to a major problem in the management of cattle farms in Kuwait. If the infected calves recover from rotavirus infection, they will suffer from health problems, e.g., loss of weight and deficiency in providing milk (12). In addition to the significant losses through calves dying, the farmers lose even more by importing replacement heifers from outside Kuwait to compensate for their shortage (12).

The present study focuses on the presence of RVA in the dairy farms of Kuwait. The occurrence of RVA in cattle is an ongoing process, because of its paramount importance for the livelihood of the cattle industry. Our study was based on the random selection of the calves, regardless of symptoms, and therefore the observed occurrence is a screenshot of the actual virus occurrence in the country.

A recent study from India showed 20.34% of diarrhoeic cattle aged 0–3 months were RVA positive (21). In another Indian study, from a different area, the diarrhoeic animals showed an RVA occurrence of 13.11% (9). In the same study the percentages of LAT and RT-PCR are comparable, which is not the case in our study. Our results show an RVA occurrence of 15.56 and 28.89%, for LAT and RT-PCR testing, respectively. The discrepancy of these methods has been observed previously (22). Most studies report a higher percentage for the LAT test and the highest specificity for the RT-PCR (23), whilst in our study LAT positive results were all confirmed by viral genome detection, resulting in a very high specificity.

Despite the known shortcomings of LAT testing, it is a useful approach because of its convenience, low price, and possibility of use in the field by non-specialized personnel (24). Nevertheless, our results showed a lot of missed animals, and thus reveal potential sources of viral spread and farm contamination. ELISA is another alternative with a good correlation to RT-PCR testing (25), but requires a laboratory setting to be conducted.

Another observation of our study was that if one animal is found positive with either technique, the possibility of having more infected animals within the same herd is quite large. It was obvious from our results that there were clusters of infection, while very few farms were virus free. We also found that one region had significantly higher occurrence of the virus than the other, however, the under-representation of the second region might account for skewed results. Overall, the detected RVA presence within our farms is quite high and further measures to contain the viral spread should be implemented (17). Vaccination of cattle has been proven to be beneficial against RVAG6 as this genotype does not appear in calves born by vaccinated mothers. Additionally, it is known that vaccination against RVA reduces the incidence of diarrhea independent of infection genotype (26). Current research has demonstrated a possible way of passive immunization of cattle with anti-bovine RVA immunoglobulin yolks (IgYs) from hens immunized with the two RVAs with different genotypes [G6P (5) and G10P (11)]. If it is confirmed, it will provide another tool against RVAs (27).

Phylogenetic analysis using concatenated NSP4, VP4, VP6 and VP7 gene sequences showed that they were all clustering within the RVA strains, having a 94% nucleotide sequence similarity. The presence of only one strain may be explained by the small size of the country and the relatively small cattle population, compared to bigger exporters and breeders worldwide. Moreover, there was a sequence affiliation with rotaviruses of human origin, suggesting a zoonotic transmission. Rotaviruses are known to have wide host range, infecting many animal species as well as humans. RVA groups A to C have been shown to infect both humans and animals. A study in Iran also highlighted the possibility of mixed rotavirus infections, in a percentage as high as 30.4% (28). A study in India, using human rotavirus specific primers, found that 2 / 230 sheep, goats and neonatal lamps fecal samples were positive for group A rotavirus (29). Given that the region with the highest occurrence in our study is known for sheep farming, we cannot exclude interspecies transmission. In Turkey, 55.33% of sheep have tested positive for RVA (30), while a similar study in Spain showed 42.6 % to harbor a mixed infection (31). The most commonly detected strains in both human and animals are G2, G3, G4, and G9, P (3).

Whilst we made sure to have a statistical validation of our sampling, nevertheless we were not able to collect samples from all dairy farms of Kuwait. Therefore, we cannot generalize our findings to the whole country. However, our results provide enough proof that rigorous preventive screening of cattle, nationwide, is imperative. Another limitation is that Kuwait is a rather small country, thus the livestock population is also limited. Big studies are therefore limited by the size of the state. The proximity of Kuwait to other large international cattle producers (e.g., India) makes our findings valuable, as trans-border infections are possible.

The present study provides baseline data for occurrence of group A rotavirus in calves under 1 year of age in Kuwait. Further in-depth studies are required to determine any correlation of the calve's viral strain with the human viral strain.

## Conclusion and Recommendations

Rotavirus is highly present all the year in calves regardless of the animal's location in Kuwait, and the virus should be included in detection and control programs by farmers and Public Authority of Agriculture and Fisheries Resources (PAAFR).

LAT could be used by farmers because of its simplicity, rapidity, and low cost when compared with molecular techniques.

Since there are advantages of LAT, it is recommended to use it for initial screening of rotavirus in dairy farms, and if there is a positive sample(s) in a farm, extend the detection using RT-PCR in all calves in the same farm to determine the precise number of calves infected with the virus.

Rotavirus screening is recommended to be in the list of pathogens by PAAFR for all dairy farms in Kuwait.

## Data Availability Statement

The datasets presented in this study can be found in online repositories. The names of the repository/repositories and accession number(s) can be found in the article/Supplementary Material.

## Ethics Statement

The animal study was reviewed and approved by Kuwait Agricultural and Fisheries Agency. Written informed consent for participation was not obtained from the owners because the authorization was taken from the Governmental Agency.

## Author Contributions

MA: project leader. AH: samples collection. SA-A, HA-A, and EH: RNA extraction, quantification, PCR amplification, gel electrophoresis, and documentation. AC, LB, and FA: performed dissemination, genetic analysis, and interpretation for viral genome. Genetic sequencing was completed in University of Leicester, UK. All authors contributed to the article and approved the submitted version.

## Funding

This research was completely funded and supported by Kuwait Institute for Scientific Research (Kuwait).

## Conflict of Interest

The authors declare that the research was conducted in the absence of any commercial or financial relationships that could be construed as a potential conflict of interest.

## Publisher's Note

All claims expressed in this article are solely those of the authors and do not necessarily represent those of their affiliated organizations, or those of the publisher, the editors and the reviewers. Any product that may be evaluated in this article, or claim that may be made by its manufacturer, is not guaranteed or endorsed by the publisher.
