# Supplementary material for: High Occurrence Among Calves and Close Phylogenetic Relationships With Human Viruses Warrants Close Surveillance of Rotaviruses in Kuwaiti Dairy Farms
Source: Front Vet Sci. 2022 Mar 8;9:745934. doi: 10.3389/fvets.2022.745934 (PMC8959109; doi:10.3389/fvets.2022.745934)
Supplement: Supplementary file 1 [file Data_Sheet_1.docx]

Rotavirus fragments genome sequence in GenBank (Mohammad Alotaibi)

**High occurrence among calves and close phylogenetic relationships with human viruses warrants close surveillance of rotaviruses in Kuwaiti dairy farms**

| **GenBank accession number** | **GenBank address** | **Gene location** |
| --- | --- | --- |
| [MH717372](https://www.ncbi.nlm.nih.gov/nuccore/MH717372) | <https://www.ncbi.nlm.nih.gov/nuccore/MH717372> | NSP1 gene, partial cds |
| [MH717373](http://safat.kisr.edu.kw/owa/redir.aspx?C=lH3iNc0Pp0gGohdYGZdhMIfuojGCCpoh9LXhOvO3WsPN977f9XzXCA..&URL=https%3a%2f%2fwww.ncbi.nlm.nih.gov%2fnuccore%2fMH717373) | <https://www.ncbi.nlm.nih.gov/nuccore/MH717373> | NSP2-like gene, complete sequence |
| [MH717374](http://safat.kisr.edu.kw/owa/redir.aspx?C=OeXaOgOkq4x3kDbtu6IhCK_PACHZgARDf78z3Qq-_WfN977f9XzXCA..&URL=https%3a%2f%2fwww.ncbi.nlm.nih.gov%2fnuccore%2fMH717374) | <https://www.ncbi.nlm.nih.gov/nuccore/MH717374> | NSP4-like gene, partial sequence |
| [MH717375](http://safat.kisr.edu.kw/owa/redir.aspx?C=MTholB2inTkHhvcNqVOmAWXOO3xLkOcY6W4aoL-ezcjN977f9XzXCA..&URL=https%3a%2f%2fwww.ncbi.nlm.nih.gov%2fnuccore%2fMH717375) | <https://www.ncbi.nlm.nih.gov/nuccore/MH717375> | NSP5-like gene, complete sequence |
| [MH717366](https://www.ncbi.nlm.nih.gov/nuccore/MH717366) | <https://www.ncbi.nlm.nih.gov/nuccore/MH717366> | VP1 capsid protein gene, partial cds |
| [MH717367](https://www.ncbi.nlm.nih.gov/nuccore/MH717367) | <https://www.ncbi.nlm.nih.gov/nuccore/MH717367> | VP2 capsid protein gene, partial cds |
| [MH717368](https://www.ncbi.nlm.nih.gov/nuccore/MH717368) | <https://www.ncbi.nlm.nih.gov/nuccore/MH717368> | VP3 capsid protein gene, partial cds |
| [MH717369](https://www.ncbi.nlm.nih.gov/nuccore/MH717369) | <https://www.ncbi.nlm.nih.gov/nuccore/MH717369> | VP4 capsid protein gene, partial cds |
| [MH717370](https://www.ncbi.nlm.nih.gov/nuccore/MH717370) | <https://www.ncbi.nlm.nih.gov/nuccore/MH717370> | VP6 capsid protein gene, partial cds |
| [MH717371](https://www.ncbi.nlm.nih.gov/nuccore/MH717371) | <https://www.ncbi.nlm.nih.gov/nuccore/MH717371> | VP7 capsid protein gene, partial cds |
